# Sequence Determination of a Novel Tripeptide Isolated from the Young Leaves of *Azadirachta indica* A. Juss

**DOI:** 10.1155/2013/629549

**Published:** 2013-02-20

**Authors:** M. Rajeswari Prabha, B. Ramachandramurty

**Affiliations:** Department of Biochemistry, PSG College of Arts & Science, Civil Aerodrome Post, Coimbatore, Tamil Nadu 641014, India

## Abstract

The neem tree has long been recognized for its unique properties, both against insects and in improving human health. Every part of the tree has been used as a traditional medicine for household remedy against various human ailments, from antiquity. Although the occurrence of various phytochemicals in neem has been studied, we have identified the presence of a novel tripeptide in the young leaves of neem using a simple and inexpensive paper chromatographic method, detected by Cu(II)-ninhydrin reagent. The peptide nature of the isolated compound is confirmed by spectral studies. The sequence of the peptide is determined using de novo sequencing by tandem MS after purification.

## 1. Introduction

Small alpha peptides are the most expensive substances, and most of them are not easily available commercially [1]. Pharmacological studies have proved that many peptides, including those isolated from plants, have a potential antitumor effect [2]. These peptides have a number of advantages over other chemical agents including their low molecular weight, relatively simple structure, lower antigenicity, fewer adverse actions, easy absorption, and a variety of routes of administration [3]. Many antibacterial peptide families have been isolated from plants. Pp-thionin, for example, showed activity against *Rhizobium meliloti, Xanthomonas campestris*, *Micrococcus luteus*. Circulins A-B and cyclopsychotride A from the cyclotides family showed antibacterial effects against human pathogens such as *Staphylococcus aureus, Micrococcus luteus, Escherichia coli, Pseudomonas aeruginosa, Proteus vulgaris, *and *Klebsiella oxytoca *at micromolar concentrations [4]. Various plant extracts are reported to exhibit high antifungal activity due to proteins or peptides [5]. Cardiovascular activity of milk casein-derived tripeptides has also been reported, where bioactive tripeptide-containing milk products attenuated the blood pressure development in spontaneously hypertensive rats [6]. Research on *A. indica *has revealed the occurrence of various compounds such as terpenoids, and flavonoids [7, 8]. But the presence of small alpha peptides has not been reported so far.

Ninhydrin reactions using manual and automated techniques as well as ninhydrin spray reagents are widely used to analyze and characterize amino acids, peptides, and proteins, as well as numerous other ninhydrin-positive compounds in biomedical, clinical, food, forensic, histochemical, microbiological, nutritional, and plant studies [9–11]. Many of the shortcomings of ninhydrin have been met by the synthesis of a variety of ninhydrin analogs. All amino acids and their carboxyl group derivatives like esters and amides, including small peptides, produce a purple color with the classical ninhydrin reagent. This reagent was modified by us by adding cupric ion in order to distinguish qualitatively the carboxyl group derivatives of amino acids from the amino acids on paper after chromatography [12]. Amino acids produce a pink color, and their carboxyl derivatives like esters and amides, including small peptides, produce a yellow color with Cu(II)-ninhydrin reagent. The Cu(II)-ninhydrin method discussed here is a novel one because no other methods presently used can form two different coloured products with a single developing reagent. We have used this method for the detection and purification of amino acid derivatives from different plant products [13, 14]. In this paper, we report the isolation and sequence determination of a small alpha peptide from the young leaves of *A. indica*.

## 2. Materials and Methods

 Ninhydrin was acquired from Pierce (Rockford, IL, USA). Cupric nitrate was of BDH, analytical grade (Mumbai, India). Organic solvents and acids used were of the highest purity available. Whatman No. 1 filter paper discs were obtained from Whatman International Ltd, Maidstone, England. Polyvinylpyrrolidone was purchased from Loba Chemie Pvt. Ltd., Mumbai. 

### 2.1. Preparation of the Crude Extract

The young leaves of *A. indica* were homogenized (1 g/10 mL) with warm 80% ethanol. The extract was filtered through a Whatmann No. 1 filter paper. The extraction was partitioned three times with equal volumes of petroleum ether to remove the pigments. The pigment-free alcohol fraction was evaporated to dryness over a boiling water bath. The resulting residue was treated with 2% polyvinylpyrrolidone (one mL for each gram of leaf used as a starting material) and centrifuged at 3000 g for 10 min at 4°C to remove the phenolic compounds. The clear supernatant obtained was used as the crude source of the small alpha peptide.

### 2.2. Preparation of Cu(II)-Ninhydrin Reagent

 The Cu(II)-ninhydrin reagent was prepared by dissolving cupric nitrate (25 mmol/L) and ninhydrin (1% w/v) in a minimum quantity of a mixture of water and glacial acetic acid (3 : 1 v/v) and diluted with required amount of acetone.

### 2.3. Circular Paper Chromatography

The crude extract was spotted in the center of a circular Whatman No. 1 filter paper on the arc of a small circle drawn with a pencil. Depending upon the number of samples to be analyzed, the paper may be demarcated. The diameter of the samples spotted was restricted to 0.5 cm by intermittent use of a hot air dryer. The sample spotting may be repeated 15 to 20 times to ensure sufficient concentration of the component to be detected. The chromatography was carried out in an isopropanol : water (4 : 1, v/v) solvent system by connecting a filter paper wick to the solvent through a hole made at the center of the circular paper. After the run which required approximately 20–40 min, the chromatogram was dried at ambient temperature for 30 min. The air-dried chromatogram was developed by spraying uniformly with Cu(II)-ninhydrin reagent followed by drying at 60°C for 10 min.

### 2.4. Purification of the Alpha Peptide

To subject the compound for various spectral studies, a simple and inexpensive purification procedure was followed. Two circular chromatograms of these compounds were run simultaneously using Whatman No. 1 filter paper discs (12 cm). One was developed with the Cu(II)-ninhydrin reagent. The corresponding region of the paper on the other chromatogram containing the Cu(II)-ninhydrin-positive compounds was cut into pieces and eluted in 80% ethanol. The compound, thus obtained, was used for conducting the spectral studies.

### 2.5. Colorimetric Determination of the Concentration of Cu(II)-Ninhydrin-Positive Compounds

1 mL of the purified Cu(II)-ninhydrin-positive compound was added with 1 mL of Cu(II)-ninhydrin reagent, and the mixture was incubated at 40°C for 5 min. The yellow color produced was read at 420 nm. The amount of these compounds was determined by using a standard graph constructed with L-glycyl glycine as the standard.

### 2.6. UV-Vis Spectrophotometry

The purified compound was scanned for its absorption properties, from 200 nm to 900 nm in a Shimadzu, UV-Vis spectrophotometer.

### 2.7. FT-IR Spectrometry

The purified compound was also subjected to FT-IR analysis using a Shimadzu model FTIR-8300 infrared spectrometer. IR spectra were scanned between 500 and 4,000 wave numbers (per centimeter). 

### 2.8. GC-MS Analysis

The purified Cu(II)-ninhydrin-positive compound was analyzed using the GC/MS instrument: Trace Ultra version 5.0 produced by Thermo. The separation conditions were as follows: DB-5 Column 30 m × 0.25 mm × 0.25 *μ*m, mobile phase helium at flow rate 1.0 mL/min, injection chamber temperature 220°C, and oven temperature starts at 80°C raised to 250°C at a rate of 8°C per minute. The ionization mode of the mass detector was at 70 eV.

### 2.9. NMR Studies

The purified Cu(II)-ninhydrin-positive compound in deuterated acetone as a solvent was subjected to ^1^H and ^13^C NMR analysis using a Bruker 500 MHz liquid-state NMR spectrometer.

### 2.10. Acid Hydrolysis of the Purified Compound and Separation by Paper Chromatography

 For this experiment, the same sample purified using circular paper chromatography technique was employed. 0.5 mL of this sample aliquot was mixed with 0.5 mL of concentrated HCl in a clean dry test tube (the final concentration of HCl is 6 N). The tube was subsequently sealed with the help of a glass blower. This was placed in an incubator at 110°C for 24 hours after which the sample was reconstituted to 0.5 mL with distilled water. The hydrolyzed sample was spotted on circular Whatman No. 1 filter paper and developed with the isopropanol : water (4 : 1 v/v) system. The chromatograms were uniformly sprayed with ninhydrin reagent and were air dried and heated at 65°C for 10 min. 

### 2.11. De Novo Sequencing Using MS/MS

Peptide sample (purified Cu(II)-ninhydrin-positive compound) was evaporated to dryness at room temperature and resuspended in 0.1% trifluoroacetic acid buffer and spotted with CHCA (alpha-cyanohydroxycinnamic acid) matrix onto MALDI plate and allowed to dry. A model 4800 Plus MALDI TOF/TOF analyzer (Applied Biosystems Inc., Foster City, CA, USA) was used for direct profiling and MS/MS fragmentation study. Acquisitions were performed in positive ion reflectron mode. MS spectra were accumulated in mass range 400–4000 *m*/*z*. Spectra are obtained for the major peptide ions in MS mode, and sequence data are obtained when the spectrometers automatically revert to MS/MS mode. MS/MS was achieved by 2 kV collision-induced dissociation (CID) in positive ion mode. De novo sequencing analysis was used to determine the primary sequence structure for peptides that are not present in currently available databases.

## 3. Results and Discussion

**Figure 1:**
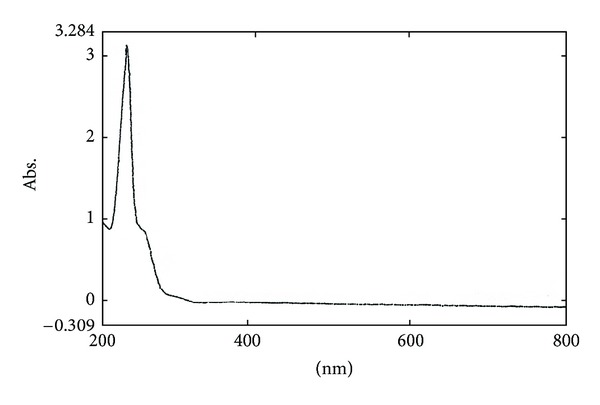
UV-Vis spectrum of the small alpha peptide purified from *A. indica. *

**Figure 2:**
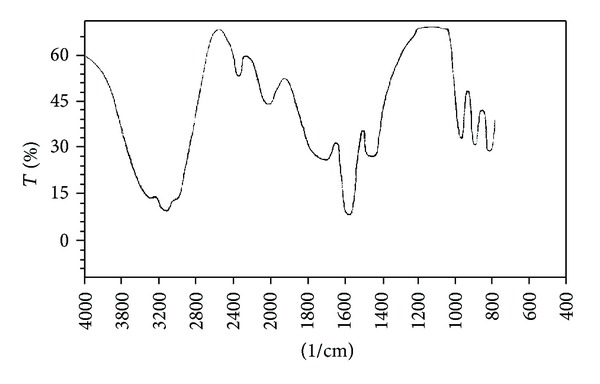
FT-IR spectrum of the small alpha peptide purified from *A. indica. *

The plant extract was run on circular paper chromatography using isopropanol : water (4 : 1) solvent system and developed with Cu(II)-ninhydrin reagent. The production of yellow chromaphore indicated the presence of Cu(II)-ninhydrin-positive compound. The detected compound was purified using inexpensive paper chromatographic method, as described. 2 *μ*g concentration of the peptide was obtained from 1 g of the leaf extract. The peptide nature of the purified compound from the young leaves of *A. indica* was confirmed by UV spectrophotometer. Purified alpha peptide from the young leaves of *A. indica *showed maximum absorption at 210 nm (Figure 1) confirming the peptide nature [15]. The peptide nature was further confirmed by identifying functional groups from the FT-IR studies.Figure 2 shows the FT-IR spectrum of the purified compound from the young leaves of *A. indica.* There were sharp peaks at 1600 cm^−1^ and 3380 cm^−1^ indicating the presence of C=O and NH groups respectively, in the compound, confirming the peptide nature [14].

**Figure 3:**
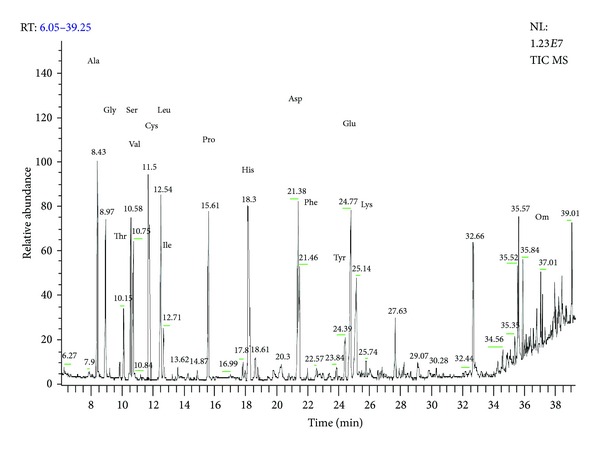
Gas chromatography retention spectra of the standard amino acids.

**Figure 4:**
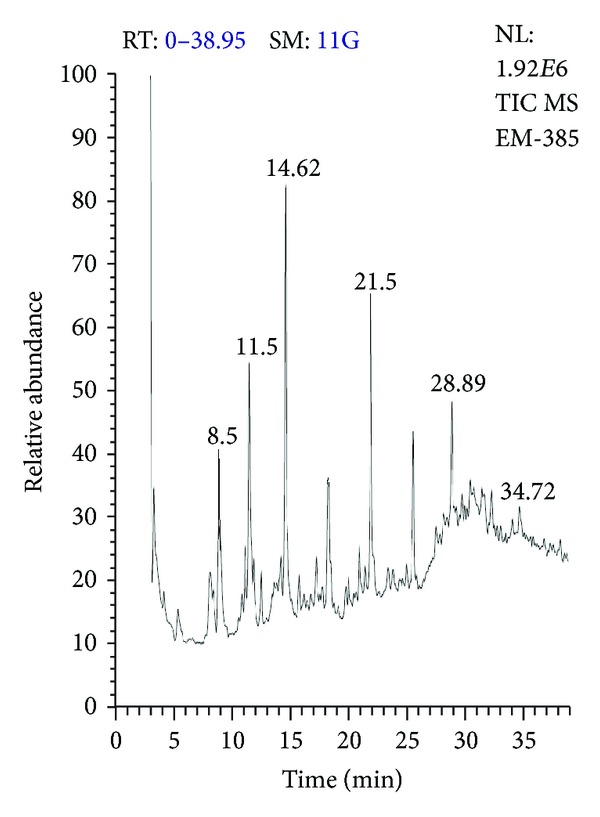
Gas chromatography retention spectra of the the Cu(II)-ninhydrin-positive compound purified from *A. indica. *

The Cu(II)-ninhydrin-positive compound purified from the young leaves of *A. indica* was analyzed by GC/MS. The retention times of the compound were compared with those of reference standard amino acids under the same conditions. The identification of the amino acids in the sample was based on direct comparison of the retention times and mass spectral data with those for standard compounds, and by computer matching with the Wiley 229, Nist 107, and 21 Library, as well as by comparison of the fragmentation patterns of the mass spectra with those reported in the literature [16]. Figure 3 shows the GC-MS spectra of the standard amino acids.  Figure 4 shows the GC-MS spectra of the Cu(II)-ninhydrin-positive compound purified from the young leaves of *A. indica*. From the data obtained, it can be inferred that the peptide isolated from the young leaves of *A. indica *might contain alanine, cysteine, and phenyl alanine.

NMR studies indicated the presence of –CH_3_, –CH_2_, and aromatic groups in proton spectra and the presence of –C=O, –CH, and SH/OH groups in carbon-13 spectra of the peptide purified from *A. indica.* This indicates the presence of aliphatic amino acid, sulphur containing amino acid, and aromatic amino acid in the isolated peptide.

**Figure 5:**
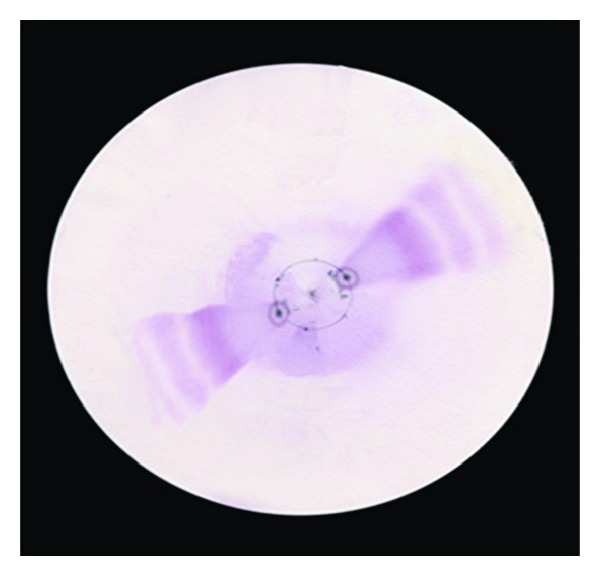
The purified small alpha peptide from *A. indica *was subjected to acid hydrolysis, spotted on a circular Whattman paper and developed using isopropanol : water (4 : 1, v/v) system and sprayed with ninhydrin reagent.

The peptide nature of the compound was also further confirmed from the acid hydrolysis experiment. The isolated compound was hydrolyzed with 6 N HCl. The chromatograms here were developed with the ninhydrin reagent which can detect the amino acids produced by hydrolysis more effectively. Figure 5 shows the acid-hydrolyzed *A. indica *peptide developed in isopropanol : water (4 : 1 v/v) system by circular paper chromatography and sprayed with ninhydrin. The result indicated that there may be presence of three amino acids in the purified peptide.

**Figure 6:**
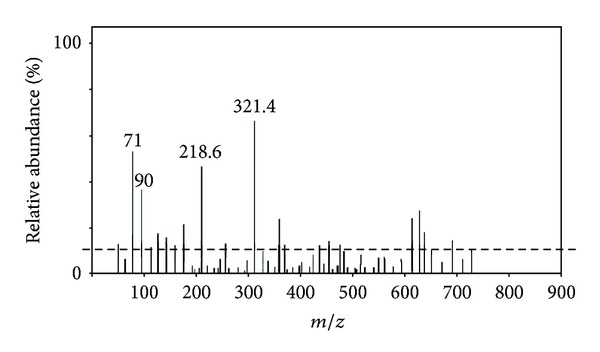
MS/MS spectra of peptide isolated from *A. indica. *

In the past decade, tandem mass spectrometry (MS/MS) has emerged as a technology of choice for high-throughput proteomics [17]. In spite of the continuously growing sequence databases, de novo sequencing of peptides, that is, sequencing without assistance of a linear sequence database, is still essential in several analytical situations. Figure 6 represents the MS/MS spectra of the peptide isolated from the young leaves of *A. indica*. Table 1 represents the amino acid sequence of the peptide isolated from *A. indica*. The sequence of amino acids was determined by the application of tandem MS. By referring to the mass unit of the respective amino acids, the amino acid sequence of the peptide isolated from the young leaves of *A. indica *is found to be Ala-Phe-Cys (N-alanine-phenylalanine-cysteine-C). The peptide isolated from *A. indica *is a tripeptide, composed of three amino acid residues with alanine at the N-terminus and cysteine at the C-terminus. The determined sequence data was submitted in UniProt Knowledgebase database, and accession number was assigned for the sequence. The sequence data of the peptide isolated from the young leaves of *A. indica*, reported in this paper, will appear in the UniProt Knowledgebase under the accession number B3EWR2.

The assessment of the biological role and applications of the purified tripeptide is under study. Compounds with low molecular weight of 500 or less can function as efficient drug molecules [18]. Small peptides containing multifunctional amino acids like L-glutamic acid, L-aspartic acid, L-lysine, L-histidine, L-cysteine, and L-serine can function as potent chelating agents that can be employed in chelation therapy [19]. Novel drugs can also be synthesised by chemical modification of these peptides.

## 4. Conclusion

From the overall results obtained from this work, it can be inferred that the Cu(II)-inhydrin positive compound purified from the young leaves of *A. indica* is a tripeptide. Unlike amino acids, small peptides are highly expensive, and most of them are not easily available commercially. Chemical synthesis of peptides increases the cost almost exponentially as the length of the peptide increases. If the separation and characterization methods for specific small peptides from inexpensive biological sources are standardized, these peptides can be easily isolated and supplied on demand for research as well as for commercial purposes. The small peptides may serve several purposes in the near future.

## Figures and Tables

**Table 1:** Sequence data summary of the peptide isolated from the young leaves of *Azadirachta indica*.

Protein mass: 321.5 Da		
Fragment ion calculator results		

Sequence: N-Ala-Phe-Cys-C		
Fragment ion table, monoisotopic masses		

Seq	No.	*B*

A	1	71.0203
F	2	218.6125
C	3	321.4521
